# Staging of transitional cell carcinoma: Has anything changed?

**DOI:** 10.4103/0970-1591.38607

**Published:** 2008

**Authors:** J. N. Kulkarni, G. K. Bakshi

**Affiliations:** Department of Uro-oncology, Asian Institute of Oncology, SL Raheja Hospital, Mahim West, Mumbai - 400 016, India

**Keywords:** Staging, transitional cell carcinioma, TNM

## Abstract

**Objective:**

This article is a systematic review of various changes in the evolution of the contemporary clinico-pathological staging of transitional cell carcinoma (TCC).

**Materials and Methods:**

A thorough search of the literature was done by Medline and other internet references.

**Results:**

Accurate staging of TCC is necessary for designing optimal therapy in clinical practice. Further, the current emphasis on bladder conservation and improved long-term disease free survival (DFS) necessitates minimal errors in staging and it's predictability towards recurrence and progression. Traditionally, the staging of TCC revolves around clinical and pathological findings. The staging has evolved through the understanding of various clinico- pathological factors like tumor appearance, number, size, grade, depth of invasion, muscle substratification, lymphovascular invasion and has reached the standard TNM classification. Cystoscopy and transurethral resection still remain the mainstay of staging and noninvasive imaging techniques have further enhanced the accuracy.

**Conclusion:**

The TNM classification for bladder cancer is currently the gold standard for TCC.

## INTRODUCTION

Urothelial cancers account for 5.6% of male and 1.8% of female cancers in India with actual crude rate (ACR) incidence of males about 1 in 174 men and 1 in 561 women.[1] Further, the epidemiology shows a strong association with environmental factors like aromatic amines, tobacco and diesel fumes. Transitional cell carcinoma (TCC) shows a spectrum of presentations from a single polypoid lesion to an invasive mass. Further, it exhibits field change potential. Clinically, these patients present with painless, intermittent hematuria.

Today, there is a consensus in dividing TCC in to two broad categories i.e. non-muscle-invasive (superficial) tumors and muscle-invasive (deep) tumors as both show distinct biological behaviors and outcomes and necessitate different therapies. The key question is can we predict accurately pathological invasion, recurrence and progression by either investigative approaches or biological markers? Various factors like depth of invasion, grade, the number of tumors and node positivity have a direct impact on the outcome of TCC. At present cystoscopy with bimanual evaluation and transurethral resection (TUR) biopsy is the standard for diagnosis and staging of TCC bladder. The aim of these procedures is to accurately stage the disease and obtain a histological diagnosis. Newer noninvasive imaging techniques like computed axial tomography (CT) (1970s) and magnetic resonance imaging (MRI) (1980s) have a definite role in detecting invasion through the bladder wall. Furthermore, the current emphasis on bladder conservation protocols require accurate staging for better results with improved quality of life.

Over the years, since noninvasive imaging modalities have not been accurate, clinical and pathological staging has been popular amongst clinicians and pathologists.

## MATERIALS AND METHODS

A thorough search of the literature was done by Medline and other internet search engines to find current pathological and clinical staging and its impact on contemporary clinical practice.

### Historical perspective

Historically, the staging of TCC has been clinico-pathological. In 1922, Broders created a landmark by formulating a grading system based on the percentage of undifferentiated urothelial cells, which was predictive of both behavior of the bladder urothelium over time and prognosis.[2]

In 1931, Aschner classified neoplasms of the bladder as papillary versus a solid configuration and in relation to the presence or absence of invasion where he found that disease severity increased with solid tumors. In 1944, Jewett and Strong analyzed the relation of depth of penetration (stage) to the incidence of local extension and metastases. In 1948, McDonald and Thompson discovered the concept of vascular and lymphatic invasion and showed that there was a direct relation to prognosis. In 1952, Jewett-Marshall-Strong redefined the staging based on bimanual palpation and biopsy into Stage 0, A and B1 (superficial disease) and B2 (deep muscle invasion) and C (2).Continuing his studies, in 1956 Marshall established the impact of gradation of tumor.

### TNM staging

**Figure 1:**
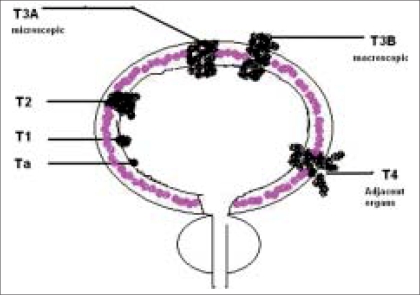
Pictorial “T” staging

Till 1967, the Jewett-Marshall-Strong classification was in vogue. However, later, Jewett and his group under the aegis of the American Joint Committee System formed the AJCC task force. This group recognized a need to broaden the staging to accommodate additional tumor characteristics and a common taxonomy. Continued efforts by this task force led to the birth of the TNM staging in 1983. The TNM classification is currently the standard staging procedure for bladder cancer and is based on clinico-pathological findings [Table 1, Figure 1].

**Table 1:** Comparison of Jewett-Strong-Marshall and TNM classification[3]

TNM	TNM	Jewett	Characteristics
Ta		0	Noninvasive papillary carcinoma
Tis			Carcinoma *in situ*: flat tumor
T1		A	Tumor invades subepithelial connective tissue (lamina propria)
T2		B	Tumor invades muscle
	T2a	B1	Tumor invades superficial muscle (inner half)
	T2b	B2	Tumor invades deep muscle (outer half)
T3		C	Tumor invades perivesical tissue
	T3a		Microscopically
	T3b		Macroscopically (extravesical mass)
T4		D1	Tumor invades any of: prostate, uterus, vagina, pelvic wall, abdominal wall
	T4a		Tumor invades prostate or uterus or vagina
	T4b		Tumor invades pelvic wall or abdominal wall
N0			No regional lymph nodes' metastasis
N1		D1	Metastasis in a single lymph node ≤2 cm in greatest dimension
N2		D1	Metastasis in a single lymph node >2 cm but ≤5 cm in greatest dimension or multiple lymph nodes none >5 cm
N3		D1	Metastasis in a lymph node >5 cm in greatest dimension single or multiple no distant metastasis
M0			No distant metastasis
M1		D2	Distant metastasis

### Pathological stages

As the clinical staging was going through changes to improve its accuracy, pathologists with the help of technical advances started redefining the tumor characteristics, grading and anatomical layers of the bladder wall like lamina propria and muscularis propria. Morphologically about 90-95% are urothelial carcinomas (UC); the ones with unusual histological appearance are called “UC with divergent differentiation”. In 1972, the World Health Organization (WHO) accepted the Mostofi stratification of TCC into three grades. Later years saw further modifications in grading due to better understanding of the biology and histological appearance. In 1997, Mostofi assembled a group of urologists, pathologists, urologic oncologists and basic scientists to make recommendations to the WHO. In 1998 WHO/ISUP (International Society of Urologists and Pathologists) proposed the concept of primary neoplastic and putative preneoplastic tumors.[4–6] Finally in 2004, the WHO formally adapted the recommendations and improvised its 2001 resolution[7,8] [Table 2]. The new classification subcategorizes superficial bladder cancer (SBC) into papilloma, PUNLMP (papillary urothelial neoplasm of low malignant potential) and TCC LG (transitional cell carcinoma low grade) corresponding to G1 and TCC HG (transitional cell carcinoma high grade) corresponding to G2 and G3.[5,6,8] T1 tumors have also been sub-stratified in to T1a, T1b and T1c based on the relation to the muscularis mucosa. However, there appears to be a great inter-observer variation amongst pathologists. Hence, Cheng et al. have tried to accurately measure the depth with the help of a micrometer.[9]

**Table 2:** Pathological classification WHO/ISUP 2004

WHO/ISUP 2004
Papilloma
PUN LMP
TCC LG
TCC HG

Vander Aa MN *et al.*, presented a new sub-staging for T1 TCC in to T1 (mic) and T1 (ext) depending on the extent of tumor invasion (< or > than 0.5 mm). Further they analyzed for mutations in the fibroblast growth factor receptor 3 (FGFR3) gene and concluded that mutant FGFR3 was commonly observed in pT1mic TCC, but rarely in pT1ext TCC. The presence of pT1ext at initial diagnosis proved to be the strongest predictor for progression, even when adjusted for FGFR3 mutation status in a Cox regression model.[10]

### Staging techniques

#### Cystourethroscopy and resection

Endoscopic evaluation of lower urinary tract includes careful inspection of the urethra with bladder. Further careful and accurate mapping of tumor/tumors with respect to site, size, relation to the bladder neck and ureteric orifices and the intervening mucosa is mandatory. Next, bimanual palpation of the bladder is done to assess the invasion of the wall of the bladder and fixity. However, it has an error of 25-50% in staging.[2] Transurethral resection of the tumor or tumors is both therapeutic as well as diagnostic. Complete resection of the tumor and deep biopsy of the muscle is mandatory. Tumor in a diverticulum is an important finding and due to absence of a muscle coat, it can be treated like early muscle-invasive disease.

### Imaging modalities (noninvasive)

The basic aim is to use noninvasive techniques like CT, MRI and ultrasound (USG) to document T, N and M status. A USG, being non invasive is done as initial workup. Timing of CT or MRI is either pre TUR or two to three weeks after TUR BT to increase the positive predictive value. A preoperative CT scan or MRI is advised in bladder cancer when we come across a broad-based, solid tumor of size 3 cm or more and in case of multiple tumors.

#### T status

Transabdominal, transrectal, transvaginal and transurethral US have all been used to stage bladder cancer. At times it is difficult to distinguish a big clot free or adherent to tumor from tumor itself. Often a small papillary lesion, tumor from lower ureteric end and minimally elevated tumors can be missed. Intravesical US is promising, as it has the potential to study the depth of penetration of tumors through the wall. However, it has not been possible to replace cystoscopy.

Refinement in CT over the last two decades has established its place in diagnosis and staging. The depth of invasion and loco-regional spread were the concerns of the clinician. New techniques in CT like multi-slice imaging and CT urography are comparable to flexible cystoscopy.[11]

Modern technical nuances in MRI as well as contrast media highlight tumor by suppressing signals from surrounding normal structures. Recent reports suggest that the submucosal layer is the one which enhances rather than the mucosal layer as was previously believed. The MRI has an accuracy of 73%; a sensitivity of 82%; and a specificity of 62%.[12] Although overall staging accuracy was only moderate, the accuracy for differentiating superficial versus invasive disease and organ-confined versus non-organ-confined disease was high. The advantages that MRI offers over CT include higher contrast resolution, multi planar imaging, accuracy of staging with smaller tumors and imaging in chronic renal failure (CRF).

#### N status

Regarding nodal disease, both CT and MRI can accurately predict nodal involvement if size is around 5-8 mm, while micro metastases are still undetectable. Positron emission tomography (PET) scan and PET CT (Positron emission tomography with computed axial tomography) are still in an evolving stage and may play an important role in the coming years.

### Restaging TURBT

It is a recent concept towards improving staging based on the fact that deep biopsy after primary TUR can be falsely negative due to technical reasons like charring or sampling of deeper tissue from peripheral area or with unreliable prior history. This also emphasizes the need of performing a TUR with a thin loop and pure cutting current. A second TUR in cases of T1 G2 and G3 is aimed at unmasking the presence of TCC in muscle layer thus upstaging the disease. This helps in identifying a subset of patients who require early aggressive therapy to improve survival. A second TUR in cases of T1G3 tumors helps in achieving a “radical resection”.

### Contemporary clinical practice and clinical experience

Currently, TCC bladder is categorized in to two subgroups; non-muscle-invasive (superficial) disease and muscle-invasive (deep) disease. Recently, the former has been stratified into three risk groups (low, intermediate and high) depending on grade, depth and number of tumors [Table 3].[8]

**Table 3:** Risk group classifications for noninvasive TCC

Risk group	Pathology
Low	Grade 1 Stage Ta
	Grade 1 Stage T 1 single tumor
	Size up to 1 cm
Intermediate	Grade 1 Stage T 1 multiple tumor
	Grade 2 Stage Ta
	Grade 2 Stage T 1 single tumor
	Size 1-3 cm
High	Grade 2 Stage T 1 multiple tumor
	Grade 3 Stage Ta
	Grade 3 Stage T 1 single tumor
	CIS association
	Size >3 cm

The low risk group is put on surveillance after complete TUR while the intermediate and the high risk groups are candidates for adjuvant intravesical therapy. Some high risk ones (T1c) would be considered for aggressive therapy viz. “early cystectomy”.

Radical cystectomy is the standard of care for non-metastatic invasive bladder tumors while a select group of invasive tumors can be managed by bladder conservation protocols.

Future awaits the role of detection of oncogene mutations like Rb, P 53 and presence of FGFR3 gene mutations in predicting the recurrences and progression.

## CONCLUSION

Over the years, the staging of TCC has been evolving due to better understanding of tumor biology and potential for progression. Noninvasive techniques of imaging have a definite role and are likely to undergo further refinements to achieve accuracy. The TNM classification for bladder cancer is successfully used all over for its uniformity, benefits, accuracy and clarity and simplicity. Cystoscopy and TURBT still remain the gold standard for diagnosis, staging and treatment. Concept of restaging TURBT is an important step ahead towards bladder preservation or early cystectomy, either of which will provide a high quality of life in the long term.

## References

[1] Kavarana NM, Kamat MR, Kurkure AP (2000). National cancer registry project.

[2] Macvicar AD (2006). Bladder cancer staging. BJU Int.

[3] Cummings (1992). Diagnosis and staging of bladder cancer, UCNA.

[4] Epstein JI, Amen MB, Reuter VR, Mostofi FK (1998). The World Health Organization/International Society of Urological Pathologists consensus classification of urothelial (transitional cell) neoplasms of urinary bladder. Am J Surg Pathol.

[5] Reuter VR, Epstein JI, Amin MB, Mostofi FK (1999). The “WHO / ISUP Consensus Classification of urothelial (transitional cell) Neoplasms”: Continued discussion: Pathology of bladder cancer: Assessment of the primary lesion and response therapy. Hum Pathol.

[6] Eble J, Santer G, Epstein J (2004). Pathology and Genetics of Tumors of the urinary system and male genital organs.

[7] Reuter VC (2006). The Pathology of bladder cancer. Urology.

[8] Millan-Rodriguez F, Chechile-Toniolo G, Salvado-Bayarri J, Palou J, Algaba F, Vicente-Rodriguez J (2000). Primary superficial bladder cancer risk groups according to progression, mortality and recurrence. J Urol.

[9] Cheng L, Weaver AL, Neumann RM, Scherer BG, Bostwick DG (1999). Sub staging of T1 bladder carcinoma based on the depth of invasion as measured by micrometer: A new proposal. Cancer.

[10] van der Aa MN, van Leenders GJ, Steyerberg EW, van Rhijn BW, Jöbsis AC, Zwarthoff EC (2005). A new system for sub staging pT1 papillary bladder cancer: A prognostic evaluation. Hum Pathol.

[11] Turney BW, Jonathan MG Willatt, Nixon D, Crew JP, Cowan NC (2006). Computed tomography urography for diagnosing bladder cancer. BJU Int.

[12] Husband JE, Olliff JF, Williams MP, Heron CW, Cherryman GR (1989). Bladder cancer: staging with CT and MR imaging. Radiology.

